# Medico-legal risks of point-of-care ultrasound: a closed-case analysis of Canadian Medical Protective Association medico-legal cases

**DOI:** 10.1186/s13089-024-00364-7

**Published:** 2024-02-23

**Authors:** Ross Prager, Derek Wu, Gary Garber, P. J. Finestone, Cathy Zang, Rana Aslanova, Robert Arntfield

**Affiliations:** 1 Division of Critical Care, Western University, London, Ontario Canada; 2 London Health Sciences Centre, Rm # D2-528, 800 Commissioners Rd. E., London, ON N6A 5W9 Canada; 3 Department of Medicine, Western University, London, Ontario Canada; 4 Department of Safe Medical Care Research, Canadian Medical Protective Association, Ottawa, ON Canada; 5 Department of Medicine, University of Ottawa, Ottawa, ON Canada

**Keywords:** Ultrasound, Medcio-legal, Legal, Patient safety, POCUS, Point-of-care ultrasound

## Abstract

**Background:**

Point-of-care ultrasound (POCUS) has become a core diagnostic tool for many physicians due to its portability, excellent safety profile, and diagnostic utility. Despite its growing use, the potential risks of POCUS use should be considered by providers. We analyzed the Canadian Medical Protective Association (CMPA) repository to identify medico-legal cases arising from the use of POCUS.

**Methods:**

We retrospectively searched the CMPA closed-case repository for cases involving diagnostic POCUS between January 1st, 2012 and December 31st, 2021. Cases included civil-legal actions, medical regulatory authority (College) cases, and hospital complaints. Patient and physician demographics, outcomes, reason for complaint, and expert-identified contributing factors were analyzed.

**Results:**

From 2012 to 2021, there were 58,626 closed medico-legal cases in the CMPA repository with POCUS determined to be a contributing factor for medico-legal action in 15 cases; in all cases the medico-legal outcome was decided against the physicians. The most common reasons for patient complaints were diagnostic error, deficient assessment, and failure to perform a test or intervention. Expert analysis of these cases determined the most common contributing factors for medico-legal action was failure to perform POCUS when indicated (7 cases, 47%); however, medico-legal action also resulted from diagnostic error, incorrect sonographic approach, deficient assessment, inadequate skill, inadequate documentation, or inadequate reporting.

**Conclusions:**

Although the most common reason associated with the medico-legal action in these cases is failure to perform POCUS when indicated, inappropriate use of POCUS may lead to medico-legal action. Due to limitations in granularity of data, the exact number of civil-legal, College cases, and hospital complaints for each contributing factor is unavailable. To enhance patient care and mitigate risk for providers, POCUS should be carefully integrated with other clinical information, performed by providers with adequate skill, and carefully documented.

## Background

Point-of-care ultrasound (POCUS) has become a core diagnostic tool for many physicians [1]. Its portability, excellent safety profile, and ability to make important diagnoses in real-time helps expedite care. The importance of POCUS has been recognized by many undergraduate and post-graduate medical institutions, and it has seen planned or actual integration into core medical curricula [2–5]. Additionally, its safety and ability to enhance patient care has led to its endorsement by multiple major clinical societies [6–10]. Despite being an invaluable tool, there is a risk that the rapid uptake of POCUS may outpace development of best practices to safeguard both patients and providers.

Medico-legal analyses can help promote patient safety and improve the quality of healthcare delivery. By analyzing medico-legal cases, we can identify patterns of error, understand the root causes of adverse events, and develop strategies to prevent future occurrences [11].

The current POCUS medico-legal literature has not identified any cases where performing diagnostic POCUS has resulted in civil-legal action [12–17]. Instead, all cases have arisen from failure to perform POCUS when clinically indicated [12–17]. These small studies are limited by sample size, non-comprehensive legal databases, and differences in legal systems between countries, leaving important questions about the medico-legal risk for physicians performing POCUS for Canadian physicians.

The objective of our study was to analyze the Canadian Medical Protective Association (CMPA) closed-case repository to identify medico-legal cases (civil-legal, college complaints, or hospital complaints) related to the use of POCUS. We describe the nature and frequency of medico-legal claims, identify common errors and contributing factors, and discuss strategies for mitigating medico-legal risks when performing POCUS.

## Methods

In January 2023, the Canadian Medical Protective Association (CMPA), a not-for-profit mutual defense organization, represented over 107,000 physician members. The CMPA offers medico-legal support, advice, and education to physicians, and engages in safe medical care research using medico-legal data from its repository. The repository relies on physician members to voluntarily contact the CMPA and submit materials when seeking advice or support for medico-legal matters. We conducted a 10-year retrospective descriptive analysis of medico-legal cases related to diagnostic POCUS performed in a hospital setting. In this study, cases included civil-legal actions (class action legal cases were excluded), medical regulatory authority cases (College) and hospital complaints. A statistical data analyst searched all cases closed between January 1st, 2012 and December 31st, 2021. Prior to analysis, all cases were de-identified and reported at the aggregate level to ensure confidentiality for both patients and healthcare providers. This means that detailed descriptions of individual cases are not possible and may leave an unavoidable lack of granularity. The Advarra Institutional Review Board provided ethical approval for this study.

CMPA medical analysts, who are experienced registered nurses with training in medico-legal research, used standardized methods to code information retrieved from medico-legal cases, including the case information, patient characteristics, health conditions, complications, peer expert criticisms classified using the CMPA’s contributing factors framework [11], patient harm classified using an in-house classification of harms, and the court ruling or final regulatory authority or hospital decisions. Peer experts are physicians retained by the parties in a legal action to interpret and provide their opinion on clinical, scientific, or technical issues surrounding the care provided. Clinical coding was applied using the International Statistical Classification of Diseases and Related Health Problems, 10th revision, Canada (ICD-10-CA), the Canadian Classification of Health Interventions, and in-house CMPA coding. To reduce misclassification, nurse-analysts conducted regular quality assurance reviews of coding electronically and as a group.

Closed cases with sufficient information involved various physician specialties caring for patients undergoing diagnostic POCUS. Some cases involved more than one physician and more than one physician specialty per case.

Cases were extracted using word search for point of care ultrasound (see Appendix A for list of terms). We excluded cases involving radiologists, obstetricians performing obstetric ultrasounds, and cardiologists performing echocardiography from the extraction and a manual review excluded cases when POCUS was not the medico-legal issue in the case. A descriptive analysis of the selected cases included an analysis of the contributing factors identified by peer experts as well as analysis of the indications for POCUS, patient harm, care settings, geographical location of care, patient demographics, physician specialty and years in practice.

We report all variables with frequencies and proportions using SAS software, version 9.4 for all statistical analyses (SAS^®^ Enterprise Guide^®^ software, Version 9.4. Cary, North Carolina: SAS Institute Inc.; 2013).

## Results

### Search

From 2012 to 2021, a total of 58,626 medico-legal cases were captured in the CMPA database. Of these, 31 cases met the database search strategy. POCUS was a contributing factor towards medico-legal action in 15/31 (48%) cases. Nine cases were college complaints, the rest were civil-legal actions (five) or hospital complaints. The medico-legal outcomes for physicians were decided against the physician in all 15 cases.

### Patient demographics and outcomes

Ten of the 15 patients were female (67%). The age ranges for patients were: 0–18 years old (2, 13%), 19–29 (3, 20%), 30–49 (7, 47%), 50–64 (2, 13%), and 65–79 (1, 7%). Most cases occurred in the emergency department (13, 87%). Thirteen of the 15 cases (87%) resulted from healthcare related harm to the patient, with 2 cases (13.3%) resulting from non-clinical issues (e.g. documentation). Of the 13 patients with healthcare related harm, 5 patients died (33%). For the remaining patients, the harm classification was mild for 4 patients (26.7%), moderate for 2 patients (13%) severe for 1 patient (7%), and 1 patient experienced no harm (7%). See Appendix 2 for the definitions of patient harm.

The most common patient safety indicators were diagnostic error (12/15, 80%), contraindicated procedure or pharmacotherapy (2/15, 13%), and injury associated with healthcare (1/15, 7%). The most common reasons for patient complaints included diagnostic error (14, 93%), deficient assessment (12, 80%), and failure to perform a test or intervention (8, 53%) (Table 1).

**Table 1:** Patient reason for complaint

Reason for complaint	Reason for complaint (n = 15)
Diagnostic error	14
Deficient assessment	12
Failure to perform test/intervention	8
Communication breakdown, patient	3
Misinterpretation of a test	2
Inadequate monitoring or follow-up	2
Failure to refer	2
Contraindicated medication/intervention	2
Communication breakdown, physicians	2
Professional misconduct	1
Premature discharge	1
Insufficient knowledge/skill	1
Inadequate documentation	1
Inadequate discharge process	1

Each case may have more than 1 reason for patient complaint

### Physician demographics

The distribution of specialties for physicians involved in the cases (n = 19) were: emergency medicine (9, 47%), resident physician (3,16%), family medicine (3, 16%), obstetrics and gynecology (1, 5%), internal medicine (1, 5%), general surgery (1, 5%), and diagnostic radiology (1, 5%). Note, cases where the POCUS provider was an obstetrician or radiologist were excluded from our search, however, these specialists may have been included in cases if they were listed as co-complainants. Eleven of 19 physicians (58%) were in practice 5 years of less; five of 19 physicians (26%) were in practice 11–20 years; three of 19 physicians (16%) were in practice 21–30 years. Nine cases (60%) occurred in large urban population centers (> 100,000 people), with 3 cases (20%) occurring in medium population centers (30,000 to 100,000 people), and 3 (20%) cases occurring in small population centers (less than 30,000 people).

### Expert contributing factor analysis

Expert analysis identified the root cause of medico-legal action for each case. They found that in 7 cases POCUS was not performed when clinically indicated. In 8 cases, POCUS was performed but there was an issue with its application: in 2 cases, inadequate skill; in 1 case, an incorrect approach used (e.g. a surface POCUS instead of an invasive modality); in 1 case, deficient reporting; in 1 case, deficient documentation;, in 1 case, inappropriate use; in 1 case, misdiagnosis; in 1 case, the expert analysis was not recorded. The lack of granularity of the data limits the ability to report how many of the above cases were civil-legal cases, college complaints, or hospital complaints.

For cases involving a diagnostic error (12 cases), 6 (50%) were related to performing POCUS, and 6 (50%) were related to not performing POCUS. For cases of deficient assessment, in one case, the physician relied on serial POCUS exams, disregarding an important physical exam finding that should have prompted additional imaging. In 4 cases (36%) a misinterpretation of the POCUS led to the failure to perform an indicated test or intervention. Notably, whereas only 1 case had inadequate documentation listed as the cause of complaint from the patient or family members, peer expert criticism identified 11 cases where inadequate documentation contributed to the medico-legal outcome. One important note is that not all documentation issues were specific to POCUS; however, failure to document POCUS findings, and failure to use standardized wording for POCUS reports were identified as contributing factors by peer review.

The contributing factor analysis of 14 cases with criticisms related to patient care (not the 1 case related to documentation), identified provider factors in 13/14 case (93%), team factors in 13/14 cases (93%), and system factors in 3/14 cases (21%). The complete list of provider, team, and system factors identified are included in Table 2; however, the most common contributing factors included failure to perform a test or intervention, deficient assessment, and misinterpretation of a test. At the team level, these were documentation issues and communication breakdown with a patient. At a system level these were related to resource issues, and protocol, policy, or procedural issues. Of note, the system level issues that arose from lack of resources were not related to lack of POCUS machine access or lack of access to POCUS infrastructure (e.g. image archiving), but rather inadequate staffing of departments in a way that contributed to patient harm.

**Table 2:** Individual, team, and system contributing factor analysis by peer experts

Individual contributing factor	# of case (n = 13)
Failure to perform test/intervention	11
Deficient assessment	11
Misinterpretation of a test	3
Insufficient knowledge/skill	2
Inappropriate/failure to transfer	2
Premature discharge	1
Inadequate monitoring or follow-up	1
Failure to read medical records	1
Facility administrative procedure, inadequate	1
Clinical procedure, inadequate	1
Professional misconduct	1
Factors related to provider's health	1
Poor decision-making regarding management	1
Failure to refer	1
Team contributing factors	# of case (n = 13)
Documentation issues	11
Communication breakdown, patient	3
Communication breakdown, physicians	1
System contributing factors	# of case (n = 2)
Resource issues	2
Protocol, policy and procedure issues	2

Each case may have more than 1 contributing factors

## Interpretation

In this analysis of closed medico-legal cases in the CMPA repository we identified 15 cases where POCUS was a contributing factor towards medico-legal action. Almost half of the cases were due to physicians failing to perform POCUS when indicated. In contrast to previous literature that had not identified POCUS cases that resulted in a medico-legal action [12–17], we found a number of cases where POCUS use resulted in a medico-legal action due to issues with provider skill, sonographic approach, reporting, documentation, misdiagnosis, and inappropriate use. All cases resulted in findings against the physician involved. Five cases involved patient death.

**Fig. 1:**
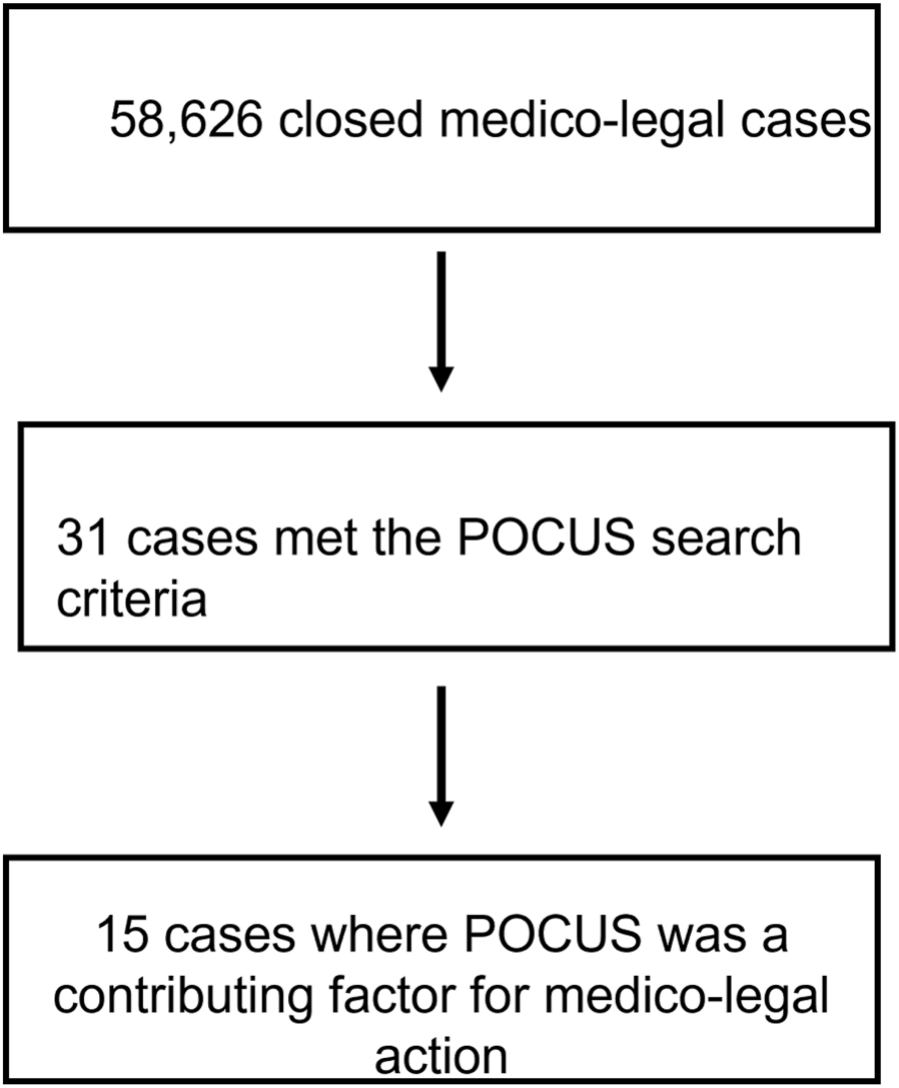
Study flow diagram

Contrasting our study to existing literature [12–17] (which report civil-legal cases only), the increased number of medico-legal cases identified may be accountable by several factors. First, the CMPA repository is a comprehensive database capturing essentially all medico-legal cases against Canadian physicians, with data coded prospectively in a highly searchable way. Additionally, most of the cases were hospital or college complaints, which have a lower barrier to filing compared with civil litigation. This is important as this is the first study to examine hospital and college complaints, in addition to civil litigation. Thus, whereas patients and families may have felt POCUS was applied inappropriately and submitted a hospital or college complaint, it may not have met a threshold to proceed with civil litigation (Fig. 1).

Regardless, our study demonstrates an important reality for the twenty-first century acute care physician: failure to perform POCUS when indicated may result in medico-legal action. For some specialties and indications, POCUS may no longer be an adjunct to augment traditional bedside assessment but rather a core part of the diagnostic process itself. While POCUS infrastructure and expertise varies between different settings, as the evidence and use of POCUS grows, so too can the expectation that it is appropriately used when clinically indicated. This may be particularly relevant for specific use cases of POCUS with well-established diagnostic pathways including the Focused Assessment with Sonography in Trauma (FAST) exam during trauma resuscitation, or its use to diagnosis undifferentiated shock [18–20]. The excellent safety and diagnostic prowess of acute care POCUS has led to its endorsement by various societal guidelines [6–10].

Despite increasing adoption of POCUS in acute care medicine, adequate training and skill is necessary for its safe implementation, and in this analysis was flagged as a contributing factor for medico-legal action in several cases. One systems level approach to POCUS education being implemented at multiple institutions is to teach ultrasound physics, knobology, and anatomy in parallel with traditional medical school curriculum to provide learners with a base skillset that can be built on during further training [3–5]. Then, specialty specific POCUS training with a focus on interpretation and synthesis can be taught during residency. Although this approach will help ensure future generations of physicians have base competency in POCUS, for clinicians in practice, alternative educational approaches should be considered. These include informal or formal instruction from colleagues with POCUS expertise to help develop core skills. Alternatively, continuing medical education opportunities like POCUS courses, conferences, rotations, or fellowships are an excellent resource, however, may not be feasible for many physicians in practice. Some societies have suggested processes for credentialing and privileging practicing physicians, which may be helpful roadmaps for interested clinicians [6].

In addition to helping clinicians obtain and interpret POCUS images, formalized training helps clinicians appropriately integrate POCUS findings with other clinical information. In fact, diagnostic errors, deficient assessments, and failure to perform other indicated tests or interventions were the most common reasons for patient complaints in our study, indicating a failure to properly integrate POCUS into the diagnostic workup of patients. Ideally, POCUS findings should be integrated into patient care with a ‘Bayesian mindset’, meaning that the positive or negative finding on POCUS helps change the post-test probability of a pathology being present. This contrasts with an oversimplified view of POCUS where the presence or absence of findings on POCUS dictates whether a disease is present. This dichotomized view of POCUS is potentially dangerous, and in our experience seen more with novice POCUS practitioners. The visual nature of the medium may lend itself to a ‘seeing is believing’ phenomenon, which can lead some clinicians to place inappropriate weight on the POCUS findings, disregarding other competing clinical information.

Inappropriate integration of POCUS into practice may also result from a failure to understand the test characteristics for POCUS in that population. For instance, FAST scan has high specificity (> 98%) to detect intraperitoneal free fluid, however only moderate sensitivity (70–90%) with test characteristics varying between operators [18, 21, 22]. For a trauma patient with a very high pre-test probability for intrabdominal hemorrhage (e.g. 80%), even with a negative FAST scan (assume sensitivity of 70%), the post-test chance of intrabdominal hemorrhage is 55%. Overreliance on POCUS and failure to integrate other clinical information is a crucial pitfall to avoid.

Inadequate documentation led to medico-legal action and was a major theme in the contributing factor analysis. If a POCUS is performed, the indication, views acquired, findings, and interpretation should be recorded in the patient's chart. At a minimum, this should be written as a progress note or included as part of a consultation. A better practice, although not available at many centers, is to save images in an accessible archiving system, and then generate a written report to allow for accountability and communication between providers [23, 24]. Ideal practice would have all archived scans undergo quality assurance by a POCUS expert, with the amended reports subsequently uploaded into a patient’s electronic medical record. The practice of "shadow" scans where results are communicated by verbal handover between providers is not acceptable and may expose physicians to medico-legal risk.

### Future directions

Although this represents a preliminary analysis of Canadian medico-legal cases involving POCUS, we expect the number of medico-legal cases to grow in parallel with increased POCUS use across. A repeat analysis of this work in 5 or 10 years will be helpful to assess for evolving patterns in POCUS medico-legal risk. Furthermore, this study excluded procedural use of POCUS (e.g. central line insertion) which would be an important area for future research. Additionally, knowledge translation surrounding best practices in POCUS training, clinical integration, and documentation is needed to promote optimal POCUS use among physicians.

### Limitations

There are several important limitations: the search terms used may not have retrieved all medico-legal cases related to POCUS. To address this, the CMPA will now prospectively identify POCUS cases to facilitate future research. We omitted granular clinical details from the cases to protect patient and physician privacy, however this limits the analysis of factors leading to poor patient outcomes. This was unavoidable and was done in close collaboration with the CMPA to adhere to their rigorous privacy mandates. We recognize that this leaves unanswered questions, but feel this study still provides actionable take homes to improve patient safety. Finally, we have not included procedural POCUS, as we would lack granularity to distinguish between medico-legal action from the procedure itself, or the POCUS use.

## Conclusions

Although failing to perform POCUS when clinically indicated remains an important contributing factor for medico-legal action, in contrast to the existing published literature, we identified cases where the application of POCUS resulted in adverse medico-legal outcomes. Due to limitations in granularity of data, the exact number of civil-legal, College cases, and hospital complaints for each contributing factor is unavailable. Overall, these cases resulted from inadequate skill, misdiagnosis, incorrect approach, deficient patient assessment, and incomplete documentation. The thoughtful and deliberate integration of POCUS into diagnostic pathways will help mitigate risk while allowing patients to experience benefits of this powerful tool.

## Data Availability

Data is available on reasonable request to the corresponding author.

## Appendix 1

CMPA repository search strategy.

Word search terms:

- FAST
- Bedside
- Bedside ultrasound
- Bedside u/s
- Point of care
- Point of care ultrasound
- Point of care u/s
- POC U/S
- POC ultrasound

## Appendix 2

Patient harm classification definitions.

The harm classification distinguishes healthcare-associated harm from other medical-legal matters through a reliable method. The system categorizes harm arising from healthcare delivery either from an inherent risk of investigation or treatment, or from three types of patient safety incidents: harmful incident, no harm incident (i.e. incident occurred but did not lead to harm), or a near miss.

**Table:**

Harm classification	Definitions
Healthcare-associated harm	Harm arising from, or associated with, plan or actions taken during the provision of healthcare, rather than an underlying disease or injury
Inherent risk	Most investigations have inherent risks. Certain complications, adverse reactions, or side-effects may occur and may be independent of who is providing the care
Harmful incident	A patient safety incident that resulted in harm to the patient
No harm incident	A patient safety incident that reached a patient but no discernible harm resulted
Near miss	A patient safety incident that did not reach the patient and therefore no harm resulted
Patient safety incident	An event of circumstance which could have resulted, or did result in unnecessary harm to a patient

Harm classification table.

**Table:**

Safety event class	Level of harm	Harm category	Patient description
HI—Harmful incident (reaches the patient)			
Harm results from the care or services provided to the patient due to failures in the processes of care or in the performance of procedures, including provider error Based on expert opinion	DE—Death	HI–DE	Unexpected death not related to the natural or expected course of the patient’s illness or underlying condition. On balance of probabilities, caused by or brought forward by the incident
	SE—Severe	HI–SE	Patient harm is symptomatic: • requiring life-saving intervention or major medical/surgical intervention, • shortening life expectancy, or • causing major temporary or permanent impact on physical, mental or social function • patient experienced enduring psychological difficulty that required specialist treatment (e.g. prolonged counselling and psychotropic medication management) *Includes previous catastrophic disability*
	MO—Moderate	HI–MO	Patient harm is symptomatic: • requiring intervention (e.g. additional operative procedure, additional therapeutic treatment), and increased length of stay, or • causing temporary or permanent impact on physical, mental, or social function • patient experienced psychological difficulty requiring treatment (e.g. counselling with FP, therapist, or psychologist)
	MI—Mild	HI–MI	Patient harm is symptomatic: • symptoms are mild • minimal or no intervention is required (e.g. extra observation, investigation, review, or minor treatment) • causing minimal (permanent or temporary) impact on physical, mental, or social function • patient experienced emotional distress that had an impact on lifestyle (e.g. mild sleep disturbances, occasional work absences)
	NO—None^†^	HI–NO	Patient harm is asymptomatic. No symptoms detected and no treatment required
	ID—Insufficient detail	HI–ID	• Insufficient information is available to evaluate the level of harm • There may be no description of any clinical outcome • There may be a patient safety incident but insufficient information to classify level of harm of the outcome
IR—Inherent risk (reaches the patient)			
A known risk associated with a particular investigation, medication, or treatment. It is the risk from undergoing a procedure in ideal conditions, performed by qualified staff using current standards, equipment and techniques* Includes non-preventable missed or delayed diagnosis Based on expert opinion	DE—Death	IR–DE	Unexpected death not related to the natural or expected course of the patient’s illness or underlying condition. Caused by or brought forward as a result of the inherent risk of the care
	SE—Severe	IR–SE	Patient harm is symptomatic: • requiring life-saving intervention or major medical/surgical intervention, • shortening life expectancy, or • causing major temporary or permanent impact on physical, mental or social function • patient experienced enduring psychological difficulty that required specialist treatment (e.g. prolonged counselling and psychotropic medication management) *Includes previous catastrophic disability*
	MO—Moderate	IR–MO	Patient harm is symptomatic: • requiring intervention (e.g. additional operative procedure, additional therapeutic treatment), and increased length of stay, or • causing temporary or permanent impact on physical, mental, or social function • patient experienced psychological difficulty, requiring treatment (e.g. counselling with FP, therapist, or psychologist)
	MI—Mild	IR–MI	Patient harm is symptomatic: • symptoms are mild • minimal or no intervention is required (e.g. extra observation, investigation, review, or minor treatment) • causing minimal (permanent or temporary) impact on physical, mental, or social function • patien t experienced emotional distress that had an impact on lifestyle (e.g. mild sleep disturbances, occasional work absences)
	NO—None^†^	IR–NO	Patient harm is asymptomatic. No symptoms detected and no treatment required. *Includes persistence of condition (e.g. myopia, carpal tunnel)*
	ID—Insufficient detail	IR–ID	• Insufficient information is available to evaluate the level of harm • There may be no description of any clinical outcome • There may be a patient safety incident but insufficient information to classify level of harm of the outcome
UN—Unknown (reaches the patient)			
Harm results from the care or services provided to the patient, but no expert opinion on file to categorize as harm arising from a harmful incident or inherent risk Usually involves cases with a contributing factor of UNKNOWN			Level of patient harm is not populated due to limited information on the file
NM—Near miss			
A patient safety incident that did not reach the patient and therefore no harm results	No—None	NM–NO	Error or capacity to cause harm was caught by an error detection barrier, or by chance, before reaching the patient
No healthcare-associated harm occurred	Level of harm	Harm category	Patient description
NA-not applicable		NA	Involves all other medical-legal matters that are solely administrative (e.g. documentation) or related conduct (e.g. manner)

Based on ASHRM’s Healthcare Associated Preventable Harm Level Classification and Classification of patient safety incidents in primary care.

^†^ Relates to a No harm incident: a patient safety incident that reached a patient but resulted in no discernable harm.

## Abbreviations

CMPA: Canadian Medical Protective Association
FAST: Focused assessment with sonography in trauma
POCUS: Point-of-care Ultrasound
